# Analytical Validation of an ELISA Assay for Maternal Autoantibody-Related Autism

**DOI:** 10.3390/diagnostics16111665

**Published:** 2026-05-28

**Authors:** Mags McInerney, Beth Hurley, Jessica Barkow, Katherine Menning, Justin Nicolace, Joseph Schauer, Judy Van de Water, E. Robert Wassman

**Affiliations:** 1 MARAbio, Inc., Salt Lake City, UT 84101, USA; mmcinerney@marabio.com; 2 Corgenix, Inc., Broomfield, CO 80020, USAjbarkow@sebia.com (J.B.); kmenning@sebia.com (K.M.); jnicolace@sebia.com (J.N.); 3 Department of Internal Medicine and UC Davis MIND Institute, University of California Davis, Sacramento, CA 95817, USA; jdschauer@health.ucdavis.edu (J.S.); javandewater@ucdavis.edu (J.V.d.W.)

**Keywords:** autism, ASD, Maternal Autoantibody-Related Autism (MARA), indirect ELISA, LDH-A, LDH-B, GAH, STI1, CRMP-1, CRMP-2, YB-1

## Abstract

**Background/Objectives:** Genetic and environmental factors during early development contribute to autism pathogenesis. Maternal autoantibodies recognizing specific fetal brain proteins can predict autism risk in a subset of cases. These antibodies cross the placenta and bind to their target antigens, which play critical roles in neurodevelopment, thereby increasing autism risk in this mechanistically defined subtype, Maternal Autoantibody-Related Autism (MARA). A multi-ELISA assay that detects maternal autoantibody combinations associated with increased autism risk has been described in the literature. This study aimed to transfer the MARA related autoantibody component assays to a clinical development laboratory for optimization and performance characterization. **Methods:** Indirect ELISA assays for eight maternal autoantibodies targeting LDH-A, LDH-B, GAH, STI1, CRMP-1, CRMP-2, NSE, and YB-1 were transferred from an academic laboratory to a clinical development laboratory for optimization and determination of the analytical performance and preliminary assay cutoff values. Standard methodologies were used to assess linearity, sensitivity, specificity, precision, and stability. Predefined validation protocols based on professional guidelines with established acceptance criteria for each parameter were followed. **Results:** Optimized ELISAs met the acceptable analytical performance criteria. All assays except one demonstrated excellent linearity when diluted with buffer or non-reactive plasma. The sensitivity analysis showed the lower limit of quantification discretely above the limit of detection, and below the preliminary population-based threshold values. Coefficients of variation for within-lot reproducibility of positive samples were <15%, with two minor exceptions. Common interfering substances, except whole human IgG, did not affect assay performance. Microtiter assay plates were stable for at least six months without significant assay drift. **Conclusions:** These maternal autoantibody assays demonstrated high sensitivity, specificity, and robustness, supporting progression to validation in CLIA-certified clinical laboratories. These assays will enable rigorous clinical evaluation of the accuracy of specific antibody combinations previously reported in the peer reviewed literature to specifically correlate with autism.

## 1. Introduction

Autism spectrum disorder (ASD) is a neurodevelopmental condition characterized by impairments in social interaction and communication, along with restricted or repetitive patterns of thought and behavior [1]. Early developmental risk for autism is shaped not only by genetics, but also by environmental factors, including dysregulation of the maternal immune system during gestation [2,3,4]. An association between aberrant levels of maternal cytokines and chemokines during pregnancy and maternal immune activation with altered neurodevelopment in offspring has been observed in humans and animals [5,6]. Independently, the maternal production of specific autoantibodies has also been linked to autism in a subset of children [7,8]. The interplay of these two paradigms of maternal immune dysregulation in the context of altered neurodevelopment is not fully understood as yet [2,3,4].

Maternal autoantibodies reactive to fetal brain proteins were first demonstrated in 2008 in the Van de Water lab at UC Davis/MIND [7]. Multiple epidemiologic research studies at the Van de Water lab at UC Davis have shown that when specific autoantibodies against eight proteins highly expressed in the developing brain are present in maternal blood in certain specified combinations (Maternal Autoantibody-Related Autism or MARA combinations), there is a highly specific association with increased likelihood of ASD in the offspring in a subset of between 10 and 24% of mothers of individuals with autism [4,7,8,9,10,11,12,13]. Originally identified as recurring bands on Western blots from mothers of children with autism, these were shown to represent maternal autoantibodies reactive to eight specific neurodevelopmentally important proteins highly expressed in the developing fetal brain [4,7,8,9,10,11,12,13]. In a study of 61 mothers of children with ASD and 102 control mothers from the CHARGE study, machine learning-based subgroup discovery identified specific combinations of maternal autoantibodies (MARA ASD-specific patterns) with over 99% accuracy as potential biomarkers of risk for a subset of up to 18% of ASD cases [7,8,9].

These findings, along with animal model studies demonstrating autism-relevant outcomes following gestational exposure to the same autoantibodies—either by passive transfer or active autoantibody production by the dams—support the likelihood that MARA autoantibodies are not merely associated biomarkers but pathogenic agents [1,14,15,16,17,18,19,20,21]. Maternal IgG autoantibodies can cross the placenta, and during early brain development in pregnancy when the blood–brain barrier is more permissive, access target antigens within the developing brain [22,23]. A recent independent comprehensive review of maternal autoantibodies heavily focused on the work of Van de Water defining MARA as well as other less well-characterized autoantibodies affirmed that maternal autoantibodies correlate with the risk of autism in children born to mothers carrying such autoantibodies and their role as potential targetable environmental risk factors for autism [24].

Biomarkers are increasingly critical to the diagnosis and management of patients in clinical research, drug development, and especially in the clinic. However, the lack of mechanistic links to the pathology underlying autism has heretofore been limiting. Furthermore, current clinical diagnosis of autism, while accurate, relies on detailed clinical-psychological evaluations, resulting in long diagnostic odysseys and relatively late definitive diagnosis, often when the child is 3–5 years old [25]. Van de Water’s research suggests the potential that maternal autoantibodies reactive to fetal brain proteins can be predictive biomarkers of a subset of autism, based on defined combinations of these autoantibodies that are highly correlated with gold-standard clinical diagnosis of autism in offspring [4,7,8,9,10,11,12,13,26]. The identification of mechanistic biomarkers makes the rigorous translation of these findings to the clinic an important and valuable path to earlier diagnosis and potential therapeutic/preventative intervention in a subset of autistic individuals.

Enzyme-linked immunosorbent assays (ELISAs) used in research studies are powerful and practical tools for the measurement of low-abundance autoantibodies. However, methodological variations in ELISA assays may introduce both systematic and random errors as well as poor clinical quality. The first step in the clinical translation of these important findings is rigorous method validation to control assay performance suitable for its intended use [27]. Feasibility assay protocols for collapsin response mediator proteins 1 and 2 (CRMP-1 and CRMP-2), guanine deaminase (GAH), stress-induced phosphoprotein 1 (STI1), neuron-specific enolase (NSE), L-lactate dehydrogenase A and B chains (LDH-A and LDH-B), and Y-box-binding protein 1 (YB-1) [28,29,30,31,32,33,34,35,36,37,38], as described in the literature [4,7,8,9,10,11,12,13], were transferred from the Van de Water lab at UC Davis to a clinical development lab. Bridging studies confirmed equivalence between these two locations as a starting point. These were then meticulously standardized and optimized in terms of recombinant protein production, protein coating concentration, sample dilution and dilutional linearity, secondary antibody (IgG-HRP conjugate) dilution, plate coat buffer reagent exchange, lot-to-lot bridging studies of three protein production lots, and accelerated stability prior to the validation studies of analytical performance for each ELISA described here.

Analytical validation protocols were guided by the College of American Pathologists (CAP) requirements using Clinical and Laboratory Standards Institute (CLSI) guidelines encompassing linearity and recovery, analytical sensitivity, precision, analytical specificity (interference), reference range with preliminary cutoffs, and stability [39,40,41,42,43]. Acceptance criteria for each parameter were predefined to validate this set of qualitative indirect ELISA autoantibody assays as laboratory developed tests as mandated under CLIA. Accordingly, this paper presents the validation protocols and analytical performance evaluations that establish the suitability and reliability of these maternal autoantibody ELISAs for potential clinical validation and use.

## 2. Materials and Methods

### 2.1. Specimen Sources

Samples used to evaluate the analytical performance of the individual ELISAs and evaluate the population distribution of these autoantibodies were from general population donors sourced from the San Diego Blood Bank (San Diego, CA, USA) under Institutional Review Board (IRB) oversight. Samples used to verify the preliminary cutoff for each assay were obtained from the Van de Water laboratory at UC Davis (Davis, CA, USA) [13]. These samples had been classified as MARA autoantibody positive or negative using the original feasibility assays developed at UC Davis. Maternal blood was collected in 8-mL acid–citrate–dextrose (BD Diagnostic, Franklin Lakes, NJ, USA) (ACD) tubes, and plasma was separated within 24 h of collection, labeled, aliquoted, and prior to use, the samples were stored at −80 °C.

### 2.2. Protein Production

Recombinant His-tagged proteins for multiple MARA autoantibody ELISAs were produced using a baculovirus expression system in *Spodoptera frugiperda* (Sf9) or *Trichoplusia ni* (Tn5) derived insect cells. The proteins used were collapsin response mediator protein 2 (CRMP-2), collapsin response mediator protein 1 (CRMP-1), guanine deaminase (GAH), stress-induced-phosphoprotein 1 (STI1), neuron-specific enolase (NSE), L-lactate dehydrogenase A chain (LDH-A), L-lactate dehydrogenase B chain (LDH-B), and Y-box-binding protein 1 (YB-1) (all proteins sourced from Expression Systems, LLC, Davis, CA, USA). Codon-optimized genes were chemically synthesized, cloned into baculovirus transfer plasmids, and used to generate recombinant virus. Following infection and expression, cells were harvested, lysed, and the proteins purified by nickel-affinity chromatography, desalted, and sterile-filtered. Protein concentration was quantified using the bicinchoninic acid (BCA) protein assay kit (Cell Signaling Technology, Danvers, MA, USA). Proteins were then aliquoted, frozen, and shipped on dry ice for use in coating 96-well ELISA plates.

### 2.3. ELISA Plate Preparation Procedure

The recombinant antigens (proteins) were diluted with standard coating buffer to appropriate concentrations. A total of 100 uL of these mixtures was added to each well of a 96-well plate, the plate sealed, and incubated overnight at 4 °C. Plates were washed on a plate washer with wash buffer. After the final wash, excess wash buffer was removed, and 200 uL/well of blocking buffer per well was added and incubated in the covered plate for 1 h at room temperature. Plates were stored at 2–8 °C until use.

### 2.4. MARA Autoantibody ELISA Procedure Methodology

Maternal blood was collected in acid–citrate–dextrose (ACD) and plasma was separated, labeled, and aliquoted. Prior to use, samples were stored at −80 °C, and were thawed at room temperature (RT), vortexed, and centrifuged at 13,000 RPM for ten minutes. The assays were performed as indirect ELISAs, in which antigenic targets were immobilized on microtiter plates and incubated with plasma potentially containing the corresponding autoantibodies. Bound autoantibodies were subsequently detected with a labeled secondary reporter antibody.

Plasma samples, calibrator, and controls were incubated in microwells coated with purified recombinant proteins. A single point calibration method was used along with a reagent blank control. Either a 1:250 or 1:500 dilution of the calibrator, controls, and plasma samples in sample diluent was prepared. A total of 100 uL of diluted calibrator, controls, and plasma samples were added to the appropriate microwells in duplicate (including the reagent blank), and the covered plate was incubated at room temperature for 90 min. Following incubation, the plate was washed four times with wash solution using an automated plate washer without allowing the wells to dry out at any time. After the removal of unbound proteins by washing, 100 uL of a previously prepared 1:10,000 dilution of a commercial goat anti-human IgG horseradish peroxidase (HRP)-conjugated antibody (Seracare, Milford, MA, USA) was added to each well to form complexes with the target protein-bound antibodies. The plate was covered again with a plate sealer and incubated for 60 min at room temperature. After incubation with the goat-anti-human IgG, plates were washed again in the same manner four times. Following another washing step, the bound enzyme–antibody conjugate was assayed by the addition of a one-component substrate solution (100 uL) containing tetramethylbenzidine (TMB) and hydrogen peroxide (H_2_O_2_) (Neogen, Lexington, KY, USA) as the chromogenic substrate and incubated for 30 min at room temperature. Stopping solution (100 uL of 0.36 N sulfuric acid) (KTEC Equipment and Supplies, Chandler, AZ, USA) was then added to each well in the same order and at the same rate as the substrate was added to stop the enzyme reaction. In positive samples, a blue color develops in the wells at an intensity proportional to the concentration of protein-specific antibodies. Results were obtained by reading the OD of each well at 450 nm in a spectrophotometer within 5 min of adding the stopping solution, using a reference of 595 nm. The % coefficient of variation (CV) for duplicate wells of the calibrator, controls, and samples with a mean OD > 0.100 should be within ±10%. For calibrator, control, or samples with a mean OD ≤ 0.100, the difference between duplicate wells should be ≤0.050 OD. Readings of the calibrator OD should preferably be ≥0.250 OD to assure that the assay is functioning properly, and the reagent blank should preferably be ≤0.150 OD. The low control should recover less than the calibrator (<100 ELISA units (EU)), and the high control should recover greater than the calibrator (>100 EU).

### 2.5. Result Calculation and Quality Control

Studies were carried out in the Corgenix development lab using calibrated equipment with reagents and materials, including samples from UC Davis and the San Diego Blood Bank, which were procured and stored according to the established procurement policies and quality system protocols. Data and reports were generated and stored according to the development lab’s quality system. All studies were run using a single calibrator close to the estimated cutoff plus one positive and one negative control. The calibrator value was normalized to 100 EU. A conversion factor (CF) was determined for each assay run by dividing the calibrator value (100 EU) by the calibrator mean OD. Control and sample ODs from the same assay were multiplied by the CF to obtain an antibody concentration value expressed in EU.

### 2.6. Study Design: Performance Evaluation Studies

#### 2.6.1. Linearity and Recovery

Linearity was determined using serially diluted blood bank samples with high and low OD values. One protein/plate lot was assessed for each ELISA. The high OD samples were diluted in a 5-point serial dilution with both a low OD sample and a non-plasma diluent. Samples from three separate dilution preparations were assayed in duplicate and the mean, standard deviation, and %CV for each sample were calculated. Recovered values were compared to the expected values using linear regression. The guideline targets used to assess the preliminary performance were a slope of ≥0.90 and recovery of +/− 10%.

#### 2.6.2. Analytical Sensitivity

Evaluation was based on guidance from CLSI EP17-A2 [39]. Analytical sensitivity refers to the lowest concentration of a substance that an assay is capable of measuring accurately in a sample. It is determined under controlled laboratory settings and focuses mainly on evaluating the assay’s technical performance. Analytical sensitivity (i.e., limits of detection and quantitation) was assessed using one lot of protein for each antigen. Limit of blank (LOB) was determined as the mean of 40 determinations using a pool of two negative samples. The limit of detection (LOD) was based on the mean of 20 determinations from a low-level sample (one high and one negative ACD plasma sample were mixed to give five low-level samples) with ten replicates run daily for two days. It was distinguished from the LOB based on the upper 95% confidence intervals for LOB and lower 95% confidence intervals for LOD. A precision profile was prepared to determine the limit of quantification (LOQ) based on the mean of 20 determinations. Pooled negative and positive ACD plasmas created precision profile levels that were measured 10 times on each of two days. The LOQ was defined as the preparation with the lowest %CV level from the precision profile results where LOQ ≥ LOD. The mean, standard deviation, %CV, and confidence interval (CI) for the LOB, LOD, and LOQ for each sample were calculated.

#### 2.6.3. Precision

Evaluation was based on guidance from CLSI EP05-A3 [40]. Precision (repeatability and reproducibility) was assessed by a 5 × 5 × 3 matrix analysis (i.e., five replicates (mean of duplicates) of all three protein/plate lots) were run once daily over five days on four ACD plasma samples. The four plasma samples had ODs that spanned the following range: (1) blood bank samples with low ODs (negative), (2) blood bank samples with high ODs, plus two admixtures of these giving (3) low and (4) moderate test points. Outliers were identified using the Grubbs test [44]. Repeatability (within run) and reproducibility (between day, within lot, between lot) %CVs were calculated. The total reproducibility %CV was determined close to the estimated cutoff using the low-level sample evaluated over 3 lots/5 days/2 replicates. Inter-operator precision was not evaluated in these studies. Targets for repeatability were 15% CV for negative samples (Level 1) and 10% CV for samples with low, medium, or high OD samples (Levels 2–4). Targets for within-lot reproducibility were 20% for negative samples (Level 1) and 15% for samples with low, medium, or high OD samples (Levels 2–4).

#### 2.6.4. Analytical Specificity (Interference Testing)

Analytical specificity is the ability of an assay to measure the intended analyte without interference or cross-reactivity from other substances in the sample, and it was evaluated in accordance with CLSI EP07-A3 guidance [41,45]. For initial interference evaluations, the following substances (concentrations) were spiked into the samples prior to the assay. One lot of protein per antigen was evaluated with one positive and one negative ACD blood bank sample for interference by hemoglobin (1000 mg/dL) (Sigma-Aldrich, St. Louis, MO, USA, intralipid (1000 mg/dL) (Sigma-Aldrich, St. Louis, MO, USA), bilirubin (conjugate and unconjugated) (40 mg/dL) (Sigma-Aldrich, St. Louis, MO, USA), rheumatoid factor (RF) (500 IU/mL) (TECO Diagnostics, Anaheim, CA), IgG (4800 mg/dL) (Sigma-Aldrich, St. Louis, MO, USA), and anti-cardiolipin IgG (a systemic lupus erythematosus [SLE]–positive antibody) (DRG International, Springfield, NJ, USA). IgG was tested in CRMP-2 and GAH only as testing was discontinued due to interference observed at all levels. Three known SLE-positive samples were then added as an additional potential interferent. Assay results for the negative control and the preliminary cutoff for each ELISA were compared to estimate the potential for interference by each substance. A total of 26 replicates of positives and negatives with and without interferents were tested (except SLE samples). Mean, standard deviation, and %CV were calculated for each condition and interferent and compared to the control value in each case. Substances were deemed non-interfering if within ±20%. If this criterion was not met, dose–response testing was performed to determine the concentration of interferent meeting the criteria.

#### 2.6.5. Stability (Plate Shelf-Life at 4 °C)

Each protein was assessed across three plate lots with four ACD plasma samples spanning the range from negative (lowest OD available) to high (highest OD available), plus two admixtures resulting in low and moderate level ODs. Two replicates (independent dilutions) were tested in duplicate at multiple timepoints. Means and standard deviations derived from precision testing were used as a guideline for the limit of allowable drift. Data for each assay at each time point were assessed versus the limit of allowable drift and the shortest shelf-life from the three lots was chosen for each assay.

### 2.7. Establishment and Verification of Preliminary Cutoff Values

Reference intervals were developed based on CLSI guidance CLSI EP28: Defining, Establishing, and Verifying Reference Intervals in the Clinical Laboratory. A general population distribution of 200 female donors at the San Diego Blood Bank, who met their health screening criteria for blood donation, were assayed. The results were analyzed to affirm a normal distribution for each autoantibody and were used to set preliminary thresholds for MARA autoantibody positivity. These were tentatively set at the mean + 2SD, mean + 3SD, and 97.5th percentile of this normal population distribution. Verification of the relative performance of these potential preliminary cutoffs was evaluated with the analytically validated ELISAs with samples (N = 24 to 29 per autoantibody) previously qualified as MARA autoantibody positive or negative according to the UC Davis feasibility ELISAs. Cutoffs were selected based on the thresholds that led to the greatest separation of positive and negative samples in the verification set. The mean + 3SD, 97.5th percentile and Youden Index (YI) were applied to the verification dataset to determine the percent agreement for each ELISA in order to assess cutoff selection.

## 3. Results

### 3.1. Assay Performance

#### 3.1.1. Linearity

**Figure 1:**
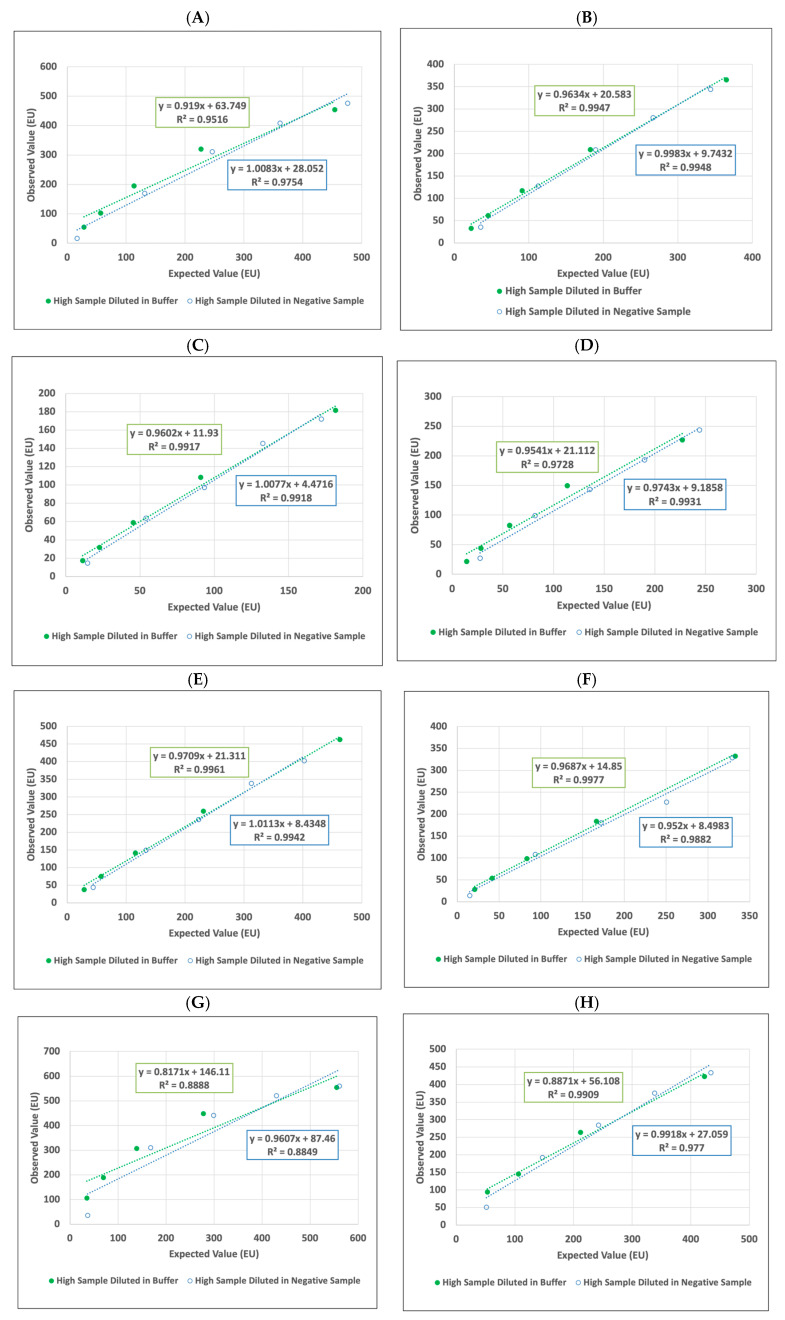
(**A**–**H**) Linearity results for each ELISA for the sample with the highest OD sample diluted in either buffer (non-plasma diluent) or negative sample (low OD ACD plasma). Linearity plots display the observed values (EU) versus the expected values (EU) for (**A**) YB-1, (**B**) CRMP-2, (**C**) LDH-B, (**D**) LDH-A, (**E**) NSE, (**F**) STI1, (**G**) CRMP-1, and (**H**) GAH. Abbreviations: ACD, acid–citrate–dextrose; EU, ELISA units.

Overall, all assays demonstrated linearity that was acceptable for qualitative assays and sufficient to proceed with further validation. Linear regression (R^2^) analysis for both diluents for seven of the eight ELISAs was ≥0.90. The anti-CRMP1 assay’s R^2^ was 0.89/0.88 (buffer/plasma), indicating slight non-linearity in that assay. Figure 1A–H. Dilutions with non-plasma diluent compared to the negative sample (low OD ACD) plasma demonstrated an overestimation of autoantibody levels for all assays. In practice, therefore, subsequent dilution of samples above the range of the spectrophotometer were diluted with low OD plasma.

#### 3.1.2. Sensitivity

ELISAs specific to each antigen were used to determine metrics of analytical sensitivity, namely, LOB, LOD, and LOQ for each autoantibody assay (Table 1).

The mean LOB results ranged from 10.9 EU to 41.1 EU, and in each case, the 95% upper limit of CI was below the 95% lower limit of the respective LOD means, which ranged from 19.4 EU to 54.1 EU. The LOQs ranged from 32.9 EU to 61.1 EU with all CVs ≤ 10%.

The LOD for each assay, which is the lowest quantity of a substance that can be distinguished from the absence of that substance (i.e., a blank value), was below the LOQ or the lowest standard curve point that is still able to be quantified for each of the assays. This means that very low concentrations can be detected before they can be measured with acceptable accuracy and precision. All LOQs were demonstrated to be below the subsequently determined preliminary thresholds.

#### 3.1.3. Precision

Precision was assessed by the evaluation of repeatability (within run) and reproducibility (between day, within lot, between lot). Reproducibility was tested across three plate/protein lots for each ELISA. Table 2, Table 3, Table 4, Table 5, Table 6, Table 7, Table 8 and Table 9 describe the results of the precision (repeatability and reproducibility) testing of each ELISA tested across four levels using blood bank samples (1 = negative, 2 = low OD, 3 = moderate OD, and 4 = high OD).

Targets for repeatability were 15% CV for Level 1 and 10% CV for Levels 2–4. The CVs ranged from 1.8% to 21.1% with 29/32 of the results being ≤10% and 12/32 being ≤5%. The three values above 10% were all Level 1: LDH-B (21.1%), CRMP-2 (16.3%), and NSE (10.1%). Targets for within-lot reproducibility were 20% for Level 1 and 15% for Levels 2–4. The CVs for within-lot reproducibility ranged from 6.5% to 32.1% with 31/32 of the results being ≤20% and 27/32 being ≤15%. The five values above 15% were Level 1 CRMP-2 (19.7%), Level 1 LDH-B (32.1%), Level 1 YB-1(15.3%), and Levels 3 and 4 CRMP-1 (17.8% and 17.1% respectively). The magnitude of the lot-to-lot %CV indicated that reproducibility depends on the ELISA process, suggesting that some potential variability remained across the protein lots and the plate coating process. This variability can be addressed going forward with ongoing process control. Overall, the precision/reproducibility of the assays was determined to be acceptable to proceed with further validation of the ELISAs.

#### 3.1.4. Stability (Plate Shelf-Life at 4 °C)

Real-time and accelerated stability testing showed that all autoantibody plates were stable out to six months from date of preparation, showing no significant variation of results over this time period.

#### 3.1.5. Analytical Specificity (Interference)

Results of interference testing showed the assays to generally be analytically specific for clinical use with limited modifications of sample acceptance criteria. Interference testing results were evaluated following the generation and verification of the preliminary cutoff for each ELISA. EU values for each sample were assessed to determine whether they were above or below the preliminary cutoff. No interference was observed at high concentrations of the common interference test substances tested—hemoglobin, intralipid, conjugated and unconjugated bilirubin, or rheumatoid factor—in either positive or negative plasma samples. IgG concentrations intended to simulate pathological levels were tested in anti-CRMP-2 and anti-GAH ELISAs only. Testing was discontinued due to interference at all levels of IgG. Significant increase in global IgG levels in patient samples may occur in several relatively uncommon clinical settings or following therapy with immunoglobulin products. Test acceptance criteria were adjusted accordingly. Alternative testing was substituted with three known SLE-positive samples to further explore interference with specific antibodies versus globally elevated IgG. Results from the three anti-cardiolipin positive samples are presented in Table 10.

Anti-cardiolipin antibody did not interfere significantly with the ELISAs (i.e., did not cause the recovered EU to exceed the preliminary cutoff). The CRMP-2 and GAH recovered EU values exceeded the preliminary cutoffs for those two ELISAs. Since these are preliminary cutoffs and significant interference was seen with IgG, a more extensive investigation of this interference will be carried out. In addition, a limitation was added to further investigate any sample that generated a positive result on all eight ELISAs.

#### 3.1.6. Summary of Assay Performance

All eight individual autoantibody assays demonstrated satisfactory performance characteristic for transfer to a clinical laboratory for future clinical validation. Table 11 summarizes the results of the performance evaluation of each ELISA for precision, sensitivity, specificity, linearity, and assay plate stability.

### 3.2. Establishment and Verification of Preliminary Cutoffs

**Figure 2:**
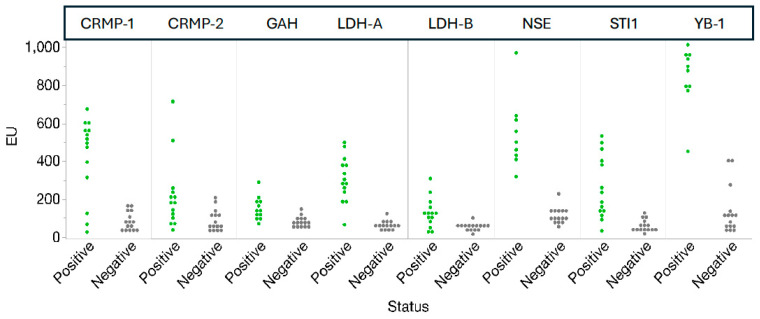
Overview of all markers by sample status. ELISA results for 24–29 clinical samples previously assigned as MARA autoantibody positive or negative. Tested using preliminary thresholds. Abbreviations: EU, ELISA units.

A set of 200 ACD plasma samples collected from healthy female donors at the San Diego Blood Bank were used to define the reference intervals and establish preliminary thresholds for each ELISA. The results approximated a normal distribution, and potential preliminary thresholds at the mean + 2 SD, mean + 3 SD, and 97.5th percentile were assessed as suitable criteria for positivity. Between 24 and 29 available clinical samples previously assigned as MARA autoantibody positive or negative by the UC Davis feasibility assays were tested using these preliminary thresholds and plotted to visualize the discrimination between autism positive and negative samples according to the UC Davis feasibility assay (Figure 2).

In addition, the Youden index (YI) for this sample set was generated using RoC (receiver operator curve) analysis to help identify cutoff thresholds that best balanced sensitivity and specificity. These YI-derived thresholds were then compared to the preliminary thresholds (97.5th percentile and mean + 3 SD) to determine which cutoff produced the optimal percent recovery and agreement with the reference assay (the UC Davis feasibility results), as summarized in Table 12.

Overall, the 97.5th percentile cutoff generated the best agreement metrics with the feasibility study results for the analytes in the verification dataset. However, in some cases, the YI improved agreement, indicating that the preliminary cutoffs could be further optimized during clinical validation. High agreement with the feasibility assay (>70%) was not always anticipated. This is because the verification assay included substantive improvements such as different protein expression methods and optimized parameters and components.

Generation of assay thresholds using a fixed method, here the 97.5th percentile of a general population distribution, led to the identification of sets of assay combinations that were similar to those identified in the academic literature [4,7,8,9,10,11,12,13]. These cutoffs are, however, preliminary in nature and will be updated based on the results of the forthcoming clinical validity study. NSE and YB-1 demonstrated the best separation (discrimination between autism positive and negative samples) at the 97.5th threshold, and CRMP-1, LDH-A, LDH-B, and STI1 also demonstrated acceptable separation at these thresholds. Lowering the threshold for GAH to the YI value improved the separation. Separation was poorest for CRMP-2 and did not show improvement using the YI.

## 4. Discussion

The Van de Water laboratory previously identified specific combinations of the maternal autoantibodies evaluated herein, which target proteins highly expressed in the developing brain, to be associated with increased likelihood of ASD in the mother’s offspring [4,7,8,9,10,11,12,13]. Their series of independent epidemiological studies demonstrating these findings in 10–24% of mothers of children with over 99% specificity suggested their potential role as risk biomarkers for a subset of autism, MARA [8,13]. Animal models support the potential that these autoantibodies are pathogenic, analogous to other neonatal autoimmune disorders [1,14,15,16,17,18,19,20,21].

ELISA, the clinical workhorse for the assessment of antibodies, is prone to imprecision for a variety of reasons, and analytical error can be reduced by the validation strategies employed in this study. The importance of verifying the analytical performance of each individual autoantibody assay in multiplex tests intended to transfer into the clinical laboratory is critical to assure the accuracy of future assays and to develop their potential clinical utility. The maternal autoantibody ELISAs evaluated here met the acceptable analytical performance criteria for linearity, sensitivity, specificity, precision, and stability based on CLSI, CAP, and CLIA guidance [39,40,41,42,43]. The minor deviations in performance relative to pre-determined targets were all within acceptable tolerance limits for qualitative ELISAs. These ELISAs can now be transferred into a CLIA and CAP certified clinical laboratory for clinical validation to demonstrate that the previously reported performance metrics of the academic lab assays can be replicated prior to clinical use [42,43].

The analytical validation presented here of these now standardized qualitative indirect ELISA IgG autoantibodies supports their use as robust and reproducible clinical- grade assays. This is a critical first step in translating these academic discoveries into a CLIA laboratory, where large-scale clinical validation of their previously reported high predictive potential in a subset of autism may have important value in accelerating the path to diagnosis, a more accurate prediction of recurrence risk, and potentially aid in the development of interventions specific to this maternal autoimmune mechanism.

A key limitation of the current study is that since this is the first blood test for maternal plasma to assess the risk of autoimmune-related autism, no reference method or gold standard exists. Therefore, accuracy can only be assessed based on a clinical diagnosis of autism. No well-defined clinical sub-phenotype of autism has been identified to date, which further limits the ability to assess the real potential of these assays until an independent clinical validation, which is now underway, is completed.

Most experts believe that early intervention in autistic children has a significant impact on subsequent development [46,47,48,49,50,51,52]. An unmet need in autism is a test providing a quantifiable early risk indication, which may enable a shorter timeline to clinical interventions, and hopefully improve autism outcomes. Mothers with an existing autistic child who are interested in another pregnancy may also benefit from a pre-pregnancy risk assessment assay that more specifically predicts the recurrence risk, which is currently estimated empirically at approximately 20% [53]. Although these assays are not currently validated for use in pregnant women, the potential exists to improve informed decision-making prior to the conception of another pregnancy if the reported performance of autoantibody combinations for risk prediction is confirmed in clinical studies. The assay standardization and the analytical validation of the component autoantibody ELISAs as described here are a critical step prior to validation in an independent CLIA laboratory. Since many families simply opt to stop reproducing after an affected child [54], a tool for better prediction of the recurrence risk would be of potential value.

Another unmet need in autism is the identification of discrete, potentially targetable pathogenic mechanisms. The pathogenic mechanism whereby maternal auto/alloantibodies cross the placenta and target related fetal and/or newborn antigens causing neonatal autoimmune disorders is well-described, such as in lupus related congenital heart block [55], neonatal myasthenia gravis [56,57], and hemolytic disease of the fetus and newborn [58,59]. New strategies to remove such autoantibodies, block their transmission, or otherwise mitigate their impact are now a reality and might be clinically applicable to reducing the potential adverse effects on neurodevelopment by MARA autoantibodies. If the predictive value in subsequent clinical validation is confirmed, a test combining the individual autoantibody ELISAs analytically validated here may evolve into a companion diagnostic for potential interventions. IVIG, which has been broadly and safely used with varying benefits in relevant settings, may have future applications, including prenatally, for primary infertility and recurrent pregnancy loss as well as in modulating autism related antibodies that are not maternally transferred [60,61,62,63,64,65,66,67,68]. Alternatively, an emerging broad class of monoclonal antibodies targeting neonatal fragment crystallizable (Fc)-receptors (FcRn) can block the transfer of maternal alloantibodies to the fetus [68,69]. Several FcRn blockers are already being used to treat autoimmune disorders, such as myasthenia gravis [57], and more are in clinical trials, including in pregnancy for hemolytic disease of the fetus and newborn, now in a Phase III trial [58,59], where it appears more effective than IVIG. Additional strategies can potentially selectively target and degrade these maternal autoantibodies before they are transferred to the fetus during pregnancy [68,69,70,71].

In summary, the successful optimization and analytical characterization of these MARA-associated maternal autoantibody assays represent an important step toward the development of a clinically actionable tool for the identification of a subtype of autism risk. Implementation of these assays in an independent CLIA- and CAP-certified laboratory will evaluate their clinical validity, reproducibility, and potential applicability to early risk assessment and improved characterization of this autism subtype.

## Figures and Tables

**Table 1:** Sensitivity of each antigen.

Antigen	LOB (EU)	LOD (EU)	LOQ (EU) ^a^
LDH-B	10.9	19.4	57.3 (3.7% CV)
LDH-A	26.6	38.6	47.6 (5.5% CV)
YB-1	18.8	38.8	57.2 (6.8% CV)
STI1	19.0	26.6	32.9 (8.4% CV)
NSE	38.1	50.9	61.1 (10% CV)
GAH	19.0	30.8	41.1 (5% CV)
CRMP-1	31.1	46.6	56.7 (6% CV)
CRMP-2	41.1	54.1	59.4 (5% CV)

^a^ LOQ is the lowest level at which performance is acceptable; EU, ELISA units; LOB, limit of blank; LOD, limit of detection; LOQ, limit of quantitation.

**Table 2:** YB-1 summary of precision.

Level	Mean (EU)	*n*	Repeatability		Between Day		Within Lot		Between Lot		Total Reproducibility ^a^	
			SD	%CV	SD	%CV	SD	%CV	SD	%CV	SD	%CV
1	18.0	75	1.3	7.3%	2.4	13.4%	2.8	15.3%	0.1	0.6%	2.8	15.3%
2	180.5	75	6.2	3.4%	12.0	6.6%	13.5	7.5%	0.0	0%	12.9	7.1%
3	290.7	75	10.3	3.6%	22.4	7.7%	24.7	8.5%	0.0	0%	22.7	7.8%
4	575.9	75	19.6	3.4%	62.7	10.9%	65.7	11.4%	0.0	0%	59.8	10.4%

EU, ELISA units; SD, standard deviation; CV, coefficient of variation; ^a^ Precision (total reproducibility) for a low-level sample over 3 lots/5 days/2 replicates determined close to the estimated cutoff.

**Table 3:** LDH-B summary of precision.

Level	Mean (EU)	*n*	Repeatability		Between Day		Within Lot		Between Lot		Total Reproducibility ^a^	
			SD	%CV	SD	%CV	SD	%CV	SD	%CV	SD	%CV
1	8.7	75	1.8	21.1%	2.1	24.2%	2.8	32.1%	0.2	1.9%	2.8	32.2%
2	147.2	75	7.6	5.2%	16.3	11.1%	18.0	12.2%	0.0	0.0%	16.4	11.2%
3	193.7	75	12.6	6.5%	21.5	11.1%	24.9	12.9%	0.0	0.0%	23.0	11.9%
4	233.7	75	11.5	4.9%	31.4	13.4%	33.5	14.3%	0.0	0.0%	30.6	13.1%

EU, ELISA units; SD, standard deviation; CV, coefficient of variation; ^a^ Precision (total reproducibility) for a low-level sample over 3 lots/5 days/2 replicates determined close to the estimated cutoff.

**Table 4:** LDH-A summary of precision.

Level	Mean (EU)	*n*	Repeatability		Between Day		Within Lot		Between Lot		Total Reproducibility ^a^	
			SD	%CV	SD	%CV	SD	%CV	SD	%CV	SD	%CV
1	26.3	75	1.5	5.7%	2.1	7.9%	2.6	9.7%	1.6	6.0%	3.0	11.4%
2	136.4	75	7.0	5.1%	9.7	7.1%	12.0	8.8%	7.9	5.8%	14.3	10.5%
3	222.3	75	6.5	2.9%	13.0	5.8%	14.5	6.5%	0.0	0.0%	14.0	6.3%
4	290.9	75	5.2	1.8%	20.2	6.9%	20.9	7.2%	0.8	0.3%	20.9	7.2%

EU, ELISA units; SD, standard deviation; CV, coefficient of variation; ^a^ Precision (total reproducibility) for a low-level sample over 3 lots/5 days/2 replicates determined close to the estimated cutoff.

**Table 5:** STI1 summary of precision.

Level	Mean (EU)	*n*	Repeatability		Between Day		Within Lot		Between Lot		Total Reproducibility ^a^	
			SD	%CV	SD	%CV	SD	%CV	SD	%CV	SD	%CV
1	28.6	75	1.7	5.8%	3.4	11.8%	3.8	13.2%	10.3	35.9%	10.9	38.2%
2	142.9	75	7.4	5.2%	13.2	9.2%	15.2	10.6%	1.4	1.0%	15.2	10.6%
3	189.9	75	10.9	5.7%	15.8	8.3%	19.2	10.1%	0.0	0.0%	18.8	9.9%
4	280.8	75	12.3	4.4%	23.0	8.2%	26.1	9.3%	26.6	9.5%	37.3	13.3%

EU, ELISA units; SD, standard deviation; CV, coefficient of variation; ^a^ Precision (total reproducibility) for a low-level sample over 3 lots/5 days/2 replicates determined close to the estimated cutoff.

**Table 6:** CRMP-1 summary of precision.

Level	Mean (EU)	*n*	Repeatability		Between Day		Within Lot		Between Lot		Total Reproducibility ^a^	
			SD	%CV	SD	%CV	SD	%CV	SD	%CV	SD	%CV
1	24.0	75	1.8	7.3%	2.7	11.3%	3.2	13.5%	0.0	0.0%	3.0	12.4%
2	181.5	75	6.0	3.3%	17.8	9.8%	18.8	10.4%	0.0	0.0%	17.5	9.7%
3	311.3	75	15.7	5.1%	53.0	17.0%	55.3	17.8%	0.0	0.0%	51.2	16.4%
4	589.7	75	11.3	1.9%	100.1	17.0%	100.8	17.1%	0.0	0.0%	91.7	15.6%

EU, ELISA units; SD, standard deviation; CV, coefficient of variation; ^a^ Precision (total reproducibility) for a low-level sample over 3 lots/5 days/2 replicates determined close to the estimated cutoff.

**Table 7:** CRMP-2 summary of precision.

Level	Mean (EU)	*n*	Repeatability		Between Day		Within Lot		Between Lot		Total Reproducibility ^a^	
			SD	%CV	SD	%CV	SD	%CV	SD	%CV	SD	%CV
1	30.5	75	5.0	16.3%	3.4	11.2%	6.0	19.7%	0.0	0.0%	5.8	19.2%
2	184.1	75	16.7	9.1%	13.8	7.5%	21.7	11.8%	17.2	9.4%	27.7	15.0%
3	232.1	75	14.9	6.4%	23.3	10.0%	27.7	11.9%	12.4	5.3%	30.3	13.1%
4	329.4	75	33.0	10.0%	29.4	8.9%	44.1	13.4%	41.9	12.7%	60.9	18.5%

EU, ELISA units; SD, standard deviation; CV, coefficient of variation; ^a^ Precision (total reproducibility) for a low-level sample over 3 lots/5 days/2 replicates determined close to the estimated cutoff.

**Table 8:** GAH summary of precision.

Level	Mean (EU)	*n*	Repeatability		Between Day		Within Lot		Between Lot		Total Reproducibility ^a^	
			SD	%CV	SD	%CV	SD	%CV	SD	%CV	SD	%CV
1	47.5	75	3.7	7.7%	2.4	5.0%	4.4	9.2%	1.7	3.6%	4.7	9.9%
2	132.9	75	7.6	5.7%	7.8	5.8%	10.8	8.2%	22.7	17.1%	25.1	18.9%
3	258.6	75	13.4	5.2%	14.8	5.7%	20.0	7.7%	22.2	8.6%	29.9	11.6%
4	429.7	75	26.4	6.1%	41.8	9.7%	49.4	11.5%	31.6	7.4%	58.7	13.7%

EU, ELISA units; SD, standard deviation; CV, coefficient of variation; ^a^ Precision (total reproducibility) for a low-level sample over 3 lots/5 days/2 replicates determined close to the estimated cutoff.

**Table 9:** NSE summary of precision.

Level	Mean (EU)	*n*	Repeatability		Between Day		Within Lot		Between Lot		Total Reproducibility ^a^	
			SD	%CV	SD	%CV	SD	%CV	SD	%CV	SD	%CV
1	31.5	75	3.2	10.1%	3.4	10.8%	4.6	14.7%	2.6	8.3%	5.3	16.9%
2	220.3	75	7.0	3.2%	25.2	11.4%	26.1	11.9%	27.8	12.6%	38.2	17.3%
3	271.6	75	8.0	2.9%	25.2	9.3%	26.5	9.7%	29.3	10.8%	39.5	14.5%
4	439.2	75	12.3	2.8%	39.1	8.9%	41.0	9.3%	58.8	13.4%	71.7	16.3%

EU, ELISA units; SD, standard deviation; CV, coefficient of variation; ^a^ Precision (total reproducibility) for a low-level sample over 3 lots/5 days/2 replicates determined close to the estimated cutoff.

**Table 10:** Anti-cardiolipin IgG (SLE) interference results.

Antigen	SLE Sample 1 (EU)	SLE Sample 2 (EU)	SLE Sample 3 (EU)	Preliminary Cutoff (EU)	EU SLE Samples < EU Cutoff?
LDH-B	39.2	47.0	51.7	64.4	Yes
LDH-A	71.0	86.6	82.5	241.0	Yes
YB-1	62.9	92.8	239.7	646.1	Yes
STI1	52.1	75.4	59.1	169.2	Yes
NSE	95.8	97.8	114.1	304.1	Yes
GAH	109.8	125.2	114.1	93.0	No
CRMP-1	63.8	93.7	87.9	199.6	Yes
CRMP-2	102.9	120.3	94.3	77.9	No

EU, ELISA units; SLE, systemic lupus erythematosus.

**Table 11:** Summary of performance evaluation data.

ELISA	Precision [%CV] ^a^ (Low-Level Sample)	Sensitivity [LOQ] ^b^ (LOQ < Cutoff)	Specificity ^c^	Linearity [R^2^] ^d^ Buffer/Plasma	Stability
Anti-CRMP-2	15.0% (184 EU)	59 EU (Yes)	Acceptable	0.99/0.99	6 months
Anti-CRMP-1	9.7% (182 EU)	57 EU (Yes)	Acceptable	0.89/0.88	6 months
Anti-GAH	18.9% (133 EU)	41 EU (Yes)	Acceptable	0.99/0.98	6 months
Anti-STI1	10.6% (143 EU)	33 EU (Yes)	Acceptable	0.99/0.99	6 months
Anti-NSE	17.3% (220 EU)	61 EU (Yes)	Acceptable	0.99/0.99	6 months
Anti-YB-1	7.1% (181 EU)	57 EU (Yes)	Acceptable	0.95/0.98	6 months
Anti-LDH-A	10.5% (136 EU)	48 EU (Yes)	Acceptable	0.97/0.99	6 months
Anti-LDH-B	11.2% (147 EU)	57 EU (Yes)	Acceptable	0.99/0.99	6 months

^a^ Precision (total reproducibility) for 3 lots/5 days/2 replicates determined close to the estimated cutoff; ^b^ Sensitivity/LOQ is the lowest level at which performance is acceptable; ^c^ Specificity with standard interferants, hemoglobin, bilirubin, lipid, rheumatoid factor, anti-cardiolipin antibody; ^d^ High sample diluted in buffer and in plasma, linear regression assessed; Abbreviations: CV, coefficient of variation; LOQ, limit of quantitation.

**Table 12:** Agreement of verification results using different thresholds.

ELISA	M + 3 SD	97.5th	Y1
Anti-CRMP-1	89.7%	89.7%	89.7%
Anti-GAH	59.3%	59.3%	85.2%
Anti-NSE	100%	100%	100%
Anti-LDH-B	69.0%	79.3%	86.2%
Anti-YB-1	96.0%	96.0%	100%
Anti-STI1	79.3%	79.3%	86.2%
Anti-CRMP-2	72.4%	72.4%	75.9%
Anti-LDH-A	89.7%	86.2%	96.6%

M + 3 SD, mean + 3 standard deviations; 97.5th, 97.5th percentile; Y1, Youden index.

## Data Availability

The raw data supporting the conclusions of this article will be made available by the authors on request.

## Abbreviations

The following abbreviations are used in this manuscript:

ELISA	enzyme-linked immunosorbent assay
LDH-A	L-lactate dehydrogenase A chain
LDH-B	L-lactate dehydrogenase B chain
GAH	guanine deaminase or cypin
STI1	stress-induced phosphoprotein-1
NSE	neuron-specific enolase
YB-1	Y-box-binding protein 1
CRMP-1	collapsin response mediator protein 1
CRMP-2	collapsin response mediator protein 2
LOB	limit of blank
LOD	limit of detection
LOQ	lower limit of quantification
CI	confidence interval A
CD	acid–citrate–dextrose
RF	rheumatoid factor
SLE	systemic lupus erythematosus
YI	Youden index
OD	optical density or absorbance
FcRn	neonatal fragment crystallizable (Fc)-receptors
RoC	receiver operator curve
LDT	laboratory developed test
